# Physiologically Based Toxicokinetic Modelling as a Tool to Support Risk Assessment: Three Case Studies

**DOI:** 10.1155/2012/359471

**Published:** 2012-05-09

**Authors:** Hans Mielke, Ursula Gundert-Remy

**Affiliations:** ^1^Federal Institute for Risk Assessment, Max Dohrn Strasse 8-10, 10589 Berlin, Germany; ^2^Institute for Clinical Pharmacology and Toxicology, Charité-Universitätsmedizin Berlin, Charitéplatz 1, 10117 Berlin, Germany

## Abstract

In this contribution we present three case studies of physiologically based toxicokinetic (PBTK) modelling in regulatory risk assessment. (1) Age-dependent lower enzyme expression in the newborn leads to bisphenol A (BPA) blood levels which are near the levels of the tolerated daily intake (TDI) at the oral exposure as calculated by EFSA. (2) Dermal exposure of BPA by receipts, car park tickets, and so forth, contribute to the exposure towards BPA. However, at the present levels of dermal exposure there is no risk for the adult. (3) Dermal exposure towards coumarin via cosmetic products leads to external exposures of two-fold the TDI. PBTK modeling helped to identify liver peak concentration as the metric for liver toxicity. After dermal exposure of twice the TDI, the liver peak concentration was lower than that present after oral exposure with the TDI dose. In the presented cases, PBTK modeling was useful to reach scientifically sound regulatory decisions.

## 1. Introduction

Physiologically based pharmaco-/toxico-kinetic (PBPK/PBTK) modelling has a long history. The principle has been introduced by Teorell as early as 1937 [1], but uptake and further development has been slow. Beginning in the 60s, pharmacokinetics became a constituent part in drug development. In drug development a data-rich situation is given, and studies in human beings can be performed without ethical constraints. Hence, the kinetic paradigm developed under those conditions was a top down approach, where the structure of the kinetic model using compartmental models was determined by the statistically best fit to the data [2]. The kinetic parameters were estimated out of the data. Without further information the parameters are hard to interpret in a physiological context. Physiologically based pharmacokinetic models have not been used in drug kinetics to a great extent with the exception of modelling the fate of anaesthetic drugs, where it has always been an interesting approach [3, 4]. However, in the past twenty years there is growing interest in this approach as a technique to support defining the dose which is intended to be given in the first studies in humans based on preclinical in vitro and in vivo animal study results, the so-called first dose in man [5]. Similarly in toxicology, interest is growing to apply this approach to be used in risk assessment and recently an internationally agreed document has been published on the topic [6]. Here, the typical situation for a chemical is characterized by existence of data in animal species but only few, if any experimental data in humans. For risk assessment or setting limit values (e.g., tolerated daily intake (TDI), accepted daily intake (ADI), occupational exposure limit (OEL)) the animal data have to be extrapolated to the human physiology and in addition, also to the physiology of the whole population including special subgroups. Typically, default factors have been used. A factor of ten is seen to be appropriate for the species difference between animal and man (4 for toxicokinetic differences, 2.5 for toxicodynamic differences) and a second factor of ten is applied for the interspecies differences in the human population (3.2 for toxicokinetic differences; 3.2 for toxicodynamic differences). PBTK modelling may help to derive chemical-specific assessment factors at least for the kinetic portion of the chemical-specific assessment factors [7]. On the other hand, PBTK modelling may also help to better understand the mode of action by identifying which kinetic metric is really related to the toxic effect, which is to be assessed [6].

In this contribution we present three case studies, where we used PBTK modelling in order to perform a targeted risk assessment. In two cases the uncertainty surrounding the use of default values has been reduced. In one other case, the PBTK modelling supported an outcome of the assessment which is different from the default approach and also helped to identify the relevant toxicokinetic metric, thus offering some insight into the mode of action.

The modelling procedures of the examples have already been published in detail. The aim of this paper is to show how to embed PBTK modelling into a regulatory decision making process.

## 2. Regulatory Problem, Methods, and Results: Case Studies

### 2.1. Case Study. Bisphenol A: Exposure in Newborns [8]

#### 2.1.1. Regulatory Context

Bisphenol A (BPA) is a chemical used for the production of epoxy resins and polycarbonate plastics [9, 10]. Food in contact with plastic materials is one source of human exposure. Feeding bottles from plastic materials containing BPA have been identified to be a major source for exposure to infants, including neonates, whereby the oral intake by bottle fed infants was estimated to be 11 *μ*g/kg/day (worst-case estimate), several fold higher than the oral exposure of adults by the European Food Safety Agency (EFSA) [10]. The TDI (tolerated daily intake) is 50 *μ*g/kg/day derived from animal data [10]. It should be mentioned that there is ongoing controversy about the TDI being 50 *μ*g/kg/d as derived by EFSA and other regulatory agencies. There are studies showing effects of BPA below 50 *μ*g/kg/d, and some scientists are with the opinion that these studies should be used as the basis to derive the TDI [11]. We nevertheless used the regulatory TDI of 50 *μ*g/kg/d for our modelling purposes. By definition the TDI is not thought to be relevant for children below the age of 3 months. There is, however, no health-based limit value for infants of this age available. Therefore, we used this value for risk assessment. Although EFSA raised no concern for newborns it remained open whether the impairment of glucuronidation in the newborn with a capacity of less than 10% of the adult [12] may result in increased internal exposure. It should be noted that glucuronidation accounts to 85–95.5% of the metabolic clearance in adults [13, 14].

#### 2.1.2. Why PBTK Modelling?

The exposure for the bottle-fed neonate is at 1/5 of the TDI which has to be considered in conjunction with the glucuronidation capacity of less that 10% of the normal value [12]. It is highly uncertain to which extent a minor pathway (sulfation) contributing 15–7.5% to the metabolic elimination in the adult [13, 14] may compensate for the impaired metabolic capacity of the predominant pathway (glucuronidation). In order to reduce the uncertainty, we modelled the internal exposure by a human model for children of different ages including newborns and for adults in which we implemented both metabolic pathways, and we compared the internal concentrations of children at different ages and the adult with the exposure by a dose at the TDI.

#### 2.1.3. What Has Been Done?

Starting from a PBTK model containing physiological data at different ages [15], we modified the input into the model from inhalation exposure to oral exposure. The only elimination was by metabolism. Glucuronidation was parametrized using published in vitro data on *V*_max⁡_ and *K*_m_ in hepatocytes of adult humans [16] according to the formula given in [17]. Sulfation was modelled according to the relative contribution to the metabolic clearance [13]. For the newborns and infants, we searched after information on the expression of the respective enzymes in different ages and used this information to adjust the metabolic parameters. BPA undergoes glucuronidation by the UDP-glucuronyltransferase UTG2B15 in man [18]. As age-dependent expression of this enzyme is not available we used data on UTG2B7 which is from the same UTG family and has a high degree of homology with UTG2B15 [12]. The sulfation of BPA is mediated by SULT 1A1 which is expressed already in utero at high levels similar to the adult levels [19]. Ginsberg and Rice [20] claimed that tissue BPA concentrations may be higher due to deconjugation of the metabolites in tissues. We calculated that given the low partition coefficient of the polar conjugated metabolites, less than 10% of the concentration of the conjugated metabolites will enter the tissues. Nishikawa et al. [21] demonstrated that deglucuronidation is taking place, however, only to the small extent of 4.4% in the fetus as calculated by Hengstler et al. [22]. Thus, we are with the opinion that even if assuming deconjugation of metabolites in tissues, this process can be omitted from the model because it does not increase the concentration by more than about 5%.

We modelled the concentration of the parent chemical BPA in blood at a dose of 11 *μ*g/kg/day for newborn (exposure assessment by [10]) and compared the steady state concentration in blood of this dose with the steady state concentration in blood in an adult, given the identical dose of 11 *μ*g/kg/day. In the newborn, the concentration in blood was 3.3 fold higher as compared to the adult (Table 1). The steady state concentration in blood in the newborn of the realistic worst case exposure scenario did not exceed the adult steady state concentration in blood at a dose of 50 *μ*g/kg/day which is the tolerated daily intake (TDI). However, it was only 26.2% below this value (Table 1(a)). We also modelled urinary excretion of the metabolites and determined the relative contribution of the glucuronidation versus the sulfation pathway. Table 1(b) shows that in the newborn the sulfation pathway is predominant and that with increasing age (and maturation of the glucuronide pathway [12]) excretion by sulfation pathway decreases and glucuronidation becomes the predominant pathway.

#### 2.1.4. Sources of Uncertainty

**Figure 1:**
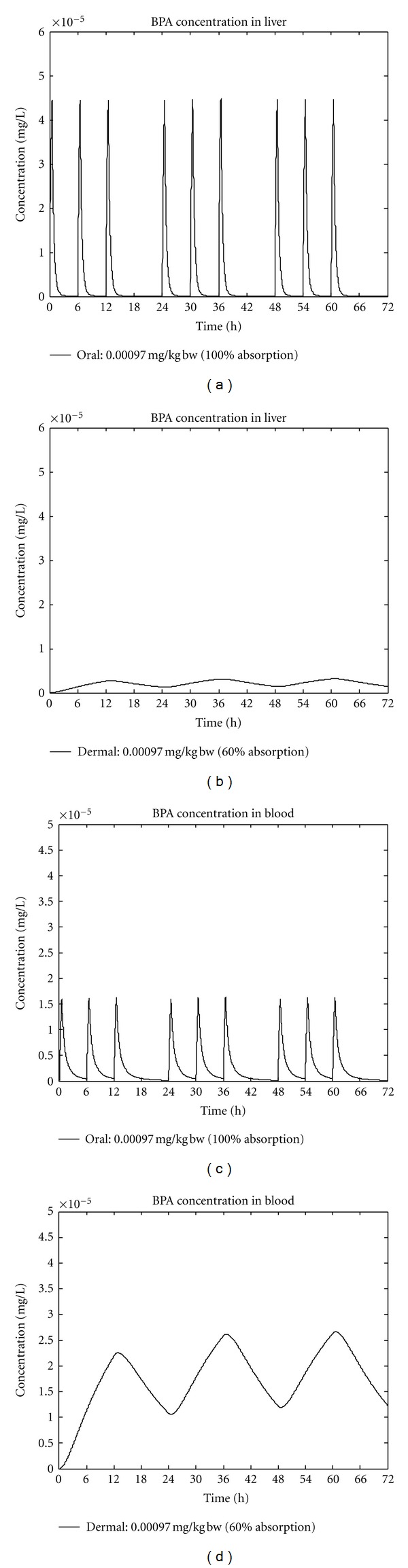
BPA: simulation results oral versus dermal route. Humans are exposed towards BPA on the oral and on the dermal routes.

Our model has several limitations. We scaled *V*_max⁡_ from in vitro the in vivo situation using a published formula which is the result of a consensus [17]. Partition coefficients were taken from experimental data in rats because data in humans were not available. The rate of absorption was estimated using the time course of urinary excretion of the conjugated metabolite which is an imprecise estimate. Finally, we assumed perfusion-limited distribution into the tissues. Concerning the age-dependent expression of UTG 2B15, we assumed that the expression pattern is the same as UTG 2B7. The data needed for validation of the model are not at hand as in experimental studies with known exposure the parent compound BPA was below the level of detection (overview in [22]). The remaining uncertainty is given by the unknown ontogeny of UTG2B15 and by the imprecise estimate of the rate of absorption. However, the simulation results were not contradictory to published simulation results, where parameter estimation has been performed differently [22]. Therefore, we do have some confidence in the results (Figure 1).

#### 2.1.5. Conclusion

The PBTK modelling results confirmed the risk assessment which has been performed on rather qualitative estimates than on quantification. However, because internal exposure expressed as the concentration in blood has been simulated for the external exposure at 11 *μ*g/kg/d in the newborn and for the exposure at the TDI for an adult, it can be seen that the oral exposure with 11 *μ*g/kg/day exhausted the TDI to 74%. Hence, it can be stated that, at oral exposure as calculated by EFSA [10], no risk is present unless exposures not accounted for so far, for example, by dermal route at high doses, would become known. As far as it is known today, there is no dermal exposure in the newborn and infant whereas dermal exposure in the adult has been found (see case study no. 2).

It is a belief that one pathway of elimination can “compensate” for a second pathway when impaired [23]. However, as shown here, although in the newborn the sulfation exceeds glucuronidation, the increased percentage eliminated via the sulfate pathway does not fully “compensate” for the impaired glucuronidation pathway which is indicated by the difference in blood levels between newborns and adults (Table 1(a)). This finding is of general importance for risk assessment in newborns and other populations at risk with impaired metabolic and renal elimination function.

### 2.2. Case Study. Bisphenol A: Dermal Exposure [24]

#### 2.2.1. Regulatory Context

 The oral route of exposure has been assumed to be the main source of exposure in consumer risk assessment of BPA [22, 24]. However, in the past several authors reported blood concentrations of BPA which were far higher than could be explained by the estimated exposure on the oral route up to now [25, 26] (citing the authors with the lowest and the highest concentrations in blood). Concerns have always been raised that the present risk assessment considering only the oral route of exposure is overlooking exposures by other routes which have to be considered to assess the true risk from BPA exposures. In 2010, several reports have been published reporting that BPA is present in thermal printing papers and products made from thermal printing paper such as receipts, car park tickets, queue tickets, ATM receipts, lottery slips, and plane, train, and bus tickets in the percentage range (0.8–3.2%) [27–29]. Furthermore, BPA is taken up on the surface of the fingers when BPA-containing paper is touched, and it is getting into the skin [27]. In [27], a daily dermal exposure of 71 *μ*g/day is estimated for the consumer, corresponding to roughly 1 *μ*g BPA/kg/day on this route.

The question is whether the additional external dermal exposure which is in the range of the external oral exposure would explain the high blood concentrations which in turn would raise concern. The TDI of 50 *μ*g/kg/day for BPA is derived from an oral study, the target organ being the liver. In the standard risk assessment the procedure is to correct the external dermal dose for the percentage of dermal absorption relative to the oral absorption. This corrected dose is then added to the oral dose. If the sum is below the TDI, no concern will be raised. As dermal exposure is a newly detected route of exposure, we applied a reverse reference scenario and estimated the dermal exposure doses necessary to yield the reported blood concentrations adding to the maximum external oral exposure estimated by FAO/WHO [30]. We did this in order to clarify whether the exposure by the dermal route can explain the high concentrations measured by some authors in blood, and whether this constitutes a concern (Table 2(b)).

#### 2.2.2. Why PBTK Modelling?

The described approach for route-to-route extrapolation is in line with the standard procedure for risk assessment. However, there are two questions. First, as the liver is the target tissue, to what extent is the liver exposed by the dermal route. Second question was to which extent is the exposure of organs other than the liver increased by dermal exposure. Given the fact that BPA has a high first pass in the liver, it is anticipated that the route of exposure is an important determinant for the concentration in organs other than the liver. A PBTK analysis was the way to tackle the problem.

#### 2.2.3. What Has Been Done?

**(1) Dermal Modelling: Risk Assessment**

We simulated the BPA concentration time profile in blood, liver, and kidney using a PBTK human model with oral route of exposure already published [8] (see above) and added a dermal pathway of exposure. The concentration time profile in kidney was simulated because minimal-to-mild nephropathy was related to doses above 50 mg/kg/day given orally in a study [31]. The extent of dermal absorption of BPA has been reported by several authors with varying values, that is, 10% [9], 13% [32], 46% [33], and 60% [27]. Based on the data of [27], we assumed that dermal absorption could be described by a diffusion process of first order and estimated a half-life of 8 hours, whereas oral absorption half-life was assumed to be 15 min as in the study of [34] the maximum concentration in the urine occurred at roughly 1 h. We performed the simulations assuming that the extent of absorption is 10%, 13%, 46%, or 60%. Here, we report only the results obtained with 60% dermal absorption. We compared the output of simulations of a dermal dose of 71 *μ*g (0.97 *μ*g/kg/day), given as a single dose, of an identical oral dose of 0.97 *μ*g/kg/day, given in three equal portions, an oral dose of 4.2 *μ*g/kg/d (FAO/WHO estimate [30]), given in three equal portions, and of 50 *μ*g/kg/day (the TDI), given in three equal portions. The results showed that dermal exposure leads to lower peak concentration in the target organ liver and to higher peak concentrations in blood and kidney as compared to the oral exposure. The AUC in blood and kidney is higher after dermal exposure as compared to dosing on the oral route. AUC in the liver is determined by the extent of absorption (Table 2(a)). With the dose of 50 *μ*g/kg/day (TDI level) by the oral route AUC in the liver is 96 fold higher. *C*_max⁡_ in the liver depends on the extent of absorption, the proportion of cardiac output which is going through the liver (22.5%) and also from the absorption half-life with slow absorption leading to low peak concentrations in the liver and fast absorption leading to high peak concentrations in the liver. *C*_max⁡_ in the liver was 700 fold higher after 50 *μ*g/kg/day (TDI level) on the oral route than after 0.97 *μ*g/kg/day by the dermal route.

**(2) Assessment of Dermal Exposure Necessary to Yield Reported Concentrations in Blood**

In addition, in order to clarify whether blood concentrations as measured by several authors were in the range of exposures measured so far we finally calculated the dermal dose, which its intake would be necessary to reach the reported concentrations of 0.33 ng/mL [25] and 5.9 ng/mL [26] when combined with the maximum oral intake of 4.2 *μ*g/kg/day as estimated by [30]. As can be seen in Table 1(b), the dermal doses of 9.4 *μ*g/kg/day and of 211.8 *μ*g/kg/day are necessary to yield concentration of 0.33 ng/mL and of 5.9 ng/mL from combined oral (as estimated by [18]) and dermal exposure, respectively.

The modeling results are in line with the physiology of dermal versus oral absorption. When absorbed through the skin, BPA first enters the venous blood. The venous blood is drained into the upper main vein, passing the right ventricle and the lungs and entering the left atrium and left ventricle. By this process the amount absorbed is mixed in the bloodstream coming from other organs. From the left ventricle, BPA is distributed via arterial blood throughout the body. In contrast, after oral administration BPA is directly delivered to the liver via the portal vein after passing through the intestinal wall. Taking all aspects together, after absorption through the skin the blood in the portal vein has a lower concentration as compared to the oral route because of the physiology. Furthermore, dermal absorption of BPA is much slower than the oral absorption which is the general rule. Thus, *C*_max⁡_ in the liver is several fold lower after dermal as compared to the oral administration.

#### 2.2.4. Source of Uncertainty

Our model has the limitations as mentioned for the BPA model above. In addition, the parameter for the rate and extent of dermal absorption as taken from the publication of Biedermann et al. [27] are approximations. The data needed to resolve this uncertainty are not easily obtained as an experimental in vivo study in humans would be necessary to be performed. Given the level of detection, we do not expect that this data need will be solved in the near future. Even in the most recent study [35] BPA concentrations were below the detection limit of 1.3 nM as analysed by CDC. Therefore, this very important point is a remaining source of uncertainty. Nevertheless, as we made worst-case assumptions the simulation results are of value to inform risk assessment.

#### 2.2.5. Conclusion

For the risk assessment, concerning liver toxicity we cannot determine what the relevant metric is. There are no data which would allow deciding whether toxicity is related to AUC or to *C*_max⁡_.

*For regulatory decision making,* the PBTK modelling and simulation results were helpful to identify a relevant route of exposure for the consumer which results in higher blood concentration than after the identical dose on the oral route. From the modeling results we can decide that the worst-case exposure estimate for consumers on the dermal route is safe.

Even if higher blood concentration of a dose given by the dermal route is taken into consideration, the doses to reach the concentrations reported by most of the authors in the literature are orders of magnitude higher than estimated, based on measurements. In this respect it should be noted that all studies in which high blood concentrations measured were uncontrolled, in particular uncontrolled in terms of the material of collecting blood, previous treatment of patients (e.g., intensive care or Cesarean section). It has been demonstrated that in intensive care the exposure towards BPA can be extremely high [36]. Hence, the high BPA concentrations measured by some authors might be explained by exposure via the intravenous route (medical devices) or by contamination when taking blood. Hence, we are with the opinion that the credibility of measured concentrations by [25, 26] is highly uncertain.

### 2.3. Case Study. Coumarin: Dermal Exposure [37]

#### 2.3.1. Regulatory Context

 Coumarin risk assessment has been performed because coumarin exposure by the oral route became a matter of concern. In addition to the oral route, humans may be exposed to coumarin by the dermal route because coumarin is used in several cosmetic products. In 2004 in an EFSA report, the risk from oral exposure by coumarin has been assessed. In this report, exposure to coumarin from cosmetic products has been mentioned being twice as high as the exposure via food [38]. Two German surveys provided detailed information on the contents of coumarin in cosmetic products [39, 40]. In the EU, there are generally agreed procedures [41–45] on how to calculate external exposure via cosmetic products based on the contents of cosmetics using default assumption on the use pattern and use frequency. Using the German data and the EU-procedures, the German Federal Institute for Risk Assessment (BfR) calculated the external coumarin exposure for a consumer by the dermal route by cosmetic products. A correction factor for skin absorption was introduced based on experimental data for route-to-route extrapolation [46, 47]. Under the assumption of a worst-case scenario the exposure level was 0.14 mg/kg bw/day which exceeds the TDI of coumarin at the level of 0.1 mg/kg bw/day and raised concern [48]. It has to be mentioned that the TDI was derived from oral studies, and that the target organ was the liver showing dose-dependent signs of toxicity.

#### 2.3.2. Why PBTK Modelling?

The risk assessment procedure is in line with the standard approach for route-to-route extrapolation. In the case of coumarin, however, the question was whether it is appropriate to use an oral TDI as a limit value to assess the risk resulting from dermal exposure or whether specific considerations apply for a substance with high first-pass elimination via hepatic metabolism such as coumarin. As there was some uncertainty concerning this question the risk assessment required further verification and substantiation. The way to solve the problem has been to perform a PBTK analysis in which it turned out that the crucial point was to identify the relevant dose metric for the toxicological endpoint which is liver toxicity.

#### 2.3.3. What Has Been Done?

**(1) PBTK Modelling of the Target Concentrations in Humans**

In humans, the kinetics of the parent compound coumarin has been studied following oral or intravenous administration of the compound [49–52]. Furthermore, in vivo and in vitro results on dermal absorption were available [44, 45]. Metabolism of coumarin was studied in in vitro experiments, and human *K*_m_ and *V*_max⁡_ values have been published [53]. The available data did allow us to use them for a human PBTK model with oral and dermal route of exposure.In the human model, we modelled absorption of 100% [50] and similar absorption half-lives (20 min for the oral and 30 min for the dermal absorption) in accordance with experimental results [46, 47, 50]. The dermal exposure to coumarin at a level of 0.1 mg/kg bw (i.e., oral TDI) resulted in a lower simulated peak concentration in the liver (*C*_max-hep_ = 1.2 *μ*g/kg liver) compared to the situation when the identical dose was given by the oral route (*C*_max-hep_ = 3.6 *μ*g/kg liver). The difference between oral *C*_max-hep_ and dermal *C*_max-hep_ increases when the rate of dermal absorption decreases. For example, in particular circumstances depending on the cosmetic preparation, as has experimentally been shown, the dermal absorption half-life is 960 min. With an extent of absorption of 100% through the skin, and this absorption half-life the peak concentration in the liver is 0.06 *μ*g/kg liver. The AUC in the liver in all cases (oral and dermal) is the same (see Table 3).The route-dependent difference of AUC in blood is explained by the fact that after oral administration the absorbed dose is undergoing first pass in the liver before entering the systemic circulation, whereas after dermal exposure the absorbed dose is undergoing first pass in the skin to a negligible extent (which we did not include into our model) before entering the systemic circulation. Hence, in this case, the systemic availability, also called bioavailability, is different from the extent of absorption. The same explanation holds true for the difference in *C*_max⁡_ in blood.The question was whether differing *C*_max⁡_ values in the liver depending on the route of administration are relevant for the risk assessment.

**(2) PBTK Modelling of Rat Data**

We identified 11 oral rat studies in the literature with information on doses and duration of the study and also information on feeding the doses (dietary or gavage). The information on dose (between 2.3 mg/kg/day and 535 mg/kg/day) and duration of exposure (between 4 weeks and 104 weeks) was used in a rat PBTK model to simulate concentration-time profiles in blood and in the liver. Rat metabolism data (*K*_m_ and *V*_max⁡_) were available from published source [53]. The resulting 31 values for the *C*_max⁡_ in the liver and AUC in the liver ranged from 0.6 to 197.1 *μ*g/g and from 529 to 590 227 *μ*g/g × h.

**(3) Assessing the Relationship between Liver Toxicity and C_max_ Versus AUC in the Liver**

In the 11 studies we identified 31 hepatotoxic responses described as the main toxicological endpoint. In each study the severity of the hepatotoxicity increased with increasing dose. We used the description of the hepatotoxic effect to grade the response into a five-point grading scale in which zero is no effect, and four is massive liver toxicity.

In order to solve the question whether the relevant toxicokinetic metric is *C*_max⁡_ or AUC in the target organ liver, we combined graded hepatic toxicity responses with *C*_max⁡_- and AUC-values in the liver as resulting from PBTK simulations in a rat model.

**Figure 2:**
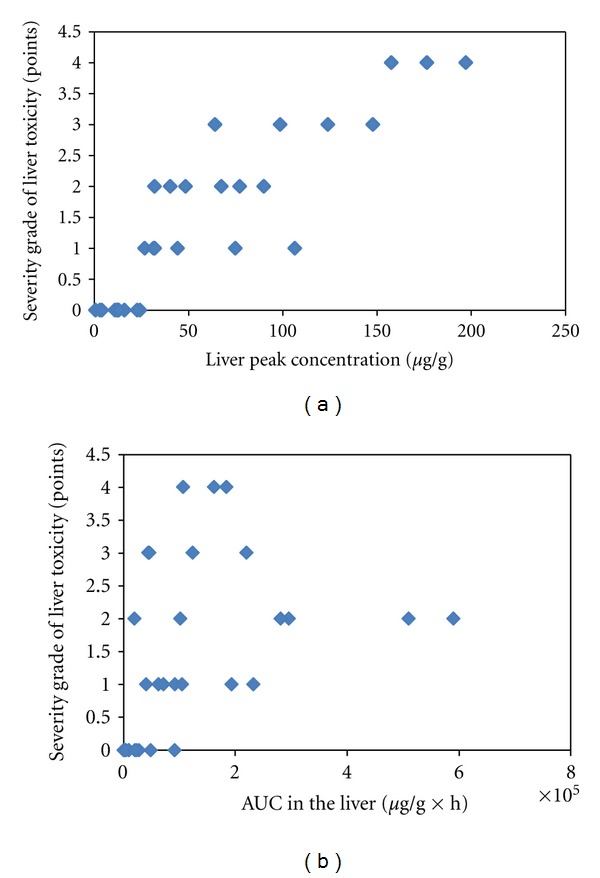
Exploration of which toxicokinetic metric is toxicodynamically relevant. Severity grade of liver toxicity (points) in relation to (a) the peak concentration in the liver (*μ*g/g liver tissue) for coumarin in rat. (b) AUC in the liver (*μ*g/g × h) for coumarin in rat. A toxicokinetic model has been constructed for the rat, and *C*_max⁡_ and AUC were simulated with doses and duration of exposure taken from published studies (*n* = 11). The toxicological endpoint in the studies was liver toxicity the degree of which differed, and we graded the toxicity in a scale from 0 to 4. *C*_max⁡_ in the liver (liver peak concentration) was better correlated to liver toxicity than AUC in the liver indicating that it *C*_max⁡_ in the liver is the toxicologically relevant toxicokinetic metric.

We performed a graphical analysis to identify whether liver toxicity was related to AUC_hep_ or to *C*_max-hep_. The analysis revealed that the severity grade of hepatotoxicity increases systematically with increasing *C*_max-hep_, whereas for AUC_hep_ no systematic increase of the severity grade with increasing AUC_hep_ could be seen (Figures 2(a) and 2(b)).

#### 2.3.4. Source of Uncertainty

Our model has the following limitations. We scaled *V*_max⁡_ from in vitro to in vivo situation using a published formula which is the result of a consensus [17]. Partition coefficients were calculated values and not experimentally obtained [5]. Although the rate and extent of absorption by the oral route and by the dermal route were taken from experimental data in humans, the data on the dermal route was estimated using the time course of urinary excretion of the conjugated metabolite which is an imprecise estimate. Finally, we assumed, perfusion limited distribution into the tissues which determines the time course in the tissue of interest, that is, the liver. The data needed to resolve this uncertainty are not easily obtained as an experimental in vivo study in humans would be necessary to be performed, in which the parent compound has to be measured. Therefore, there is remaining uncertainty. Nevertheless, when we compared the simulated oral data with the published experimental data in humans the simulation predicted the time course fairly well [37].

#### 2.3.5. Conclusion

The findings indicate that in rats coumarin-mediated liver toxicity is related to the peak liver concentration rather than to AUC in the liver. Hence, standard procedures for route-to-route extrapolation are not appropriate as they can only adjust for the amount entering the body and not for the peak concentration in the relevant organ. In conclusion, the PBTK modelling resulted in a different outcome of the risk assessment compared to the conventional approach based on external exposure or dose. As the peak concentration in the liver after dermal exposure is below the peak liver concentration resulting from oral exposure with a dose corresponding to the TDI, and peak concentration is the relevant metric, it can be stated that there is no health concern by the current exposure by cosmetic products.

As a general comment, it can be concluded that in route-to-route extrapolation for chemicals with high first-pass elimination via hepatic metabolism special attention has to be given not to underestimate the possible risk in organs other than the liver as tissue exposure (AUC) can be higher. PBTK Can help to estimate critical dose metrics (e.g., AUC and *C*_max⁡_) for various tissues within the body as a function of exposure route and intensity. While the example for coumarin shows that dermal exposures lead to lower *C*_max⁡_ of parent compound in liver as compared to an oral exposure, for compounds that have toxicities related to AUC in the liver or other target organs, dermal may lead to higher critical dose metrics. PBPK modelling helps risk assessors address these important toxicology and risk assessment issues.

## 3. General Conclusion

Risk assessment of chemicals in general and also targeted risk assessments are demanding processes. In particular, for targeted risk assessment in a regulatory environment questions have to be definitively answered. Risk assessment needs several extrapolation steps which are based on assumptions which are inherently surrounded with uncertainty. Often default assumptions have to be applied because of lack of data. However, even in cases where more data is available the preferred regulatory procedure is to apply default assumptions. In the last decade, PBTK modelling has been advocated as a means to support risk assessment and to reduce the uncertainty [6]. There is increasing awareness in regulatory decision making on the usefulness of this approach. The following examples show that BPPK modelling has found regulatory acceptance in the interspecies extrapolation from animal to man, namely, vinyl acetate, 2-butoxyethanol, propylene methyl glycol (EU Existing Chemicals Program), formaldehyde, 2-butoxyethanol (UK Health and Safety Executive), tetrachloroethylene, styrene, diethylhexlyphthalate (Health Canada), dichloromethane, ethylene glycol monobutyl ether, and vinyl chloride (US EPA (IRIS)) [54]. Cadmium is one example where human variability has been quantified, and a chemical specific factor was used instead of the default factor [54]. The cases we present in this contribution deal with scenarios, where modelling was done in human models. In the first case, the purpose was to quantify intraspecies variability for a substance, where two metabolic pathways with different maturation states in the newborn are present. In cases two and three, we elucidated the pitfalls of the conventional approach for oral to dermal extrapolation for substances with high first-pass elimination via hepatic metabolism. The three case studies demonstrate that the extrapolation using conventional approaches may lead to regulatory decisions which bear the possibility to overlook problems or to overstate the risk. In the three cases, PBTK modelling helped inform risk assessment. The answers to the questions require a physiologically appropriate structural model, knowledge on the physiological changes by life stages, and kinetics of absorption by various routes of exposure. By using modelling approaches the uncertainty is reduced. In this contribution we do not deal with uncertainty and variability in PBTK models as addressed by others for example, [55, 56]. However, we applied the lessons learned [54], and we hope that the case studies are convincing for regulators, the public, and also for scientists.

## Figures and Tables

**Table:** (a)

	Oral exposure (*μ*g/kg/day)	Steady state concentration (SSC) (ng/mL)	Percentage of TDI SSC	SSC newborn/SSC adult at 11 *μ*g/kg/day
Newborn (bottle-fed)	11 (EFSA, 2006)	0.096	73.8	3.3
Adult	11 (hypothetical)	0.029	22.3	—
Adult	50 (TDI)	0.13	100	—

**Table:** (b)

	Sulfate conjugate (percentage of the absorbed dose)	Glucuronide conjugate (percentage of the absorbed dose)
Newborn	64%	36%
3 months	31%	69%
6 months	18%	82%
1.5 year	15%	85%
Adult	15%	85%

**Table:** (a)

				Blood*		Liver		Kidney	
Route of administration	Dose (*μ*g/kg/d)	Extent of absorption (percentage of dose)	Absorption half-life (hrs)	*C*_max⁡_ (pg/g)	AUC (pg/g × h)	*C*_max⁡_ (pg/g)	AUC (pg/g × h)	*C*_max⁡_ (pg/g)	AUC (pg/g × h)
Dermal oral	0.97**	60	8	26.7	416.7	3.2	50.3	36.1	563.3
	0.97**	90	0.25	16.3	64.0	44.7	93.3	22.0	86.3
	4.2**	90	0.25	70.6	277.1	193.5	403.9	95.3	373.7
	50 (TDI)**	90	0.25	841.0	3293.3	2300	4800	1140	4433

*Blood concentration in the systemic circulation, not in the portal vein. In case of the oral route of administration, concentration in the portal vein is higher than concentration in the systemic circulation.

**Dermal dose given at once, whereas the oral doses are given in three divided portions.

**Table:** (b)

Blood concentration (mean; pg/mL)	Blood concentration of the oral dose of 4.2 *μ*g/kg/d (pg/mL)	Difference of the concentrations (pg/mL)	Dermal dose corresponding to the concentration difference (*μ*g/kg/d)
330 [25]	70.6	259.4	9.4
5900 [26]	70.6	5,829.4	211.8

**Table 3:** Comparison of the peak concentrations and AUC in blood and liver after oral and dermal exposure towards coumarin. *C*_max⁡_ and AUC of coumarin were modelled in liver and in blood after 0.1 mg/kg by the oral route (extent of absorption 100%; half-life of absorption 20 min) and dermal route (extent of absorption 100%; half-life of absorption 30 min and 960 min dependent on the cosmetic preparation). It can be seen that the AUC in the liver is identical because the amount absorbed and reaching the liver is the same. However, because of differences in the absorption half-life *C*_max⁡_ in the liver differs. In blood, AUC is different due to first pass in the liver. Even if the extent of absorption is identical the amount of coumarin reaching the systemic circulation after oral exposure is lower than after dermal exposure. *C*_max⁡_ in blood depends on the rate of absorption, expressed as half-life. If half-life of dermal absorption is similar to the oral absorption (30 min versus 20 min), *C*_max⁡_ is higher after dermal exposure (due to first pass in the liver after oral exposure and no first pass in the skin). If half-life of dermal absorption is prolonged as compared to the oral half-life of absorption (960 min versus 20 min). *C*_max⁡_ is lower. Thus, it is not only the extent but also the rate of absorption, which matters in comparing oral and dermal exposure.

Dose (mg/kg)	Route of administration	Dose fraction which is absorbed	Absorption half-life (min)	*C*_max⁡_ liver (*μ*g/kg)	AUC liver (*μ*g/kg × h)	*C*_max⁡_ blood (*μ*g/kg)	AUC blood (*μ*g/kg × h)
0.1	Oral	1.0	20	3.6	1.8	3.1	32
0.1	Dermal	1.0	30	1.2	1.8	51	77
0.1	Dermal	1.0	960	0.06	1.8	2.7	77
